# Semi-Clamshell Approach with Rib-Cross for Infected Hybrid TEVAR

**DOI:** 10.3400/avd.cr.25-00076

**Published:** 2025-10-09

**Authors:** Taiki Ito, Masato Suzuki, Shun Watanabe, Satoshi Sugimoto, Kiyotaka Morimoto, Yoshinobu Watabe, Hideo Yokoyama, Toshiro Ito

**Affiliations:** Department of Cardiovascular Surgery, Sapporo Kojinkai Memorial Hospital, Sapporo, Hokkaido, Japan

**Keywords:** semi-clamshell approach, rib-cross thoracotomy, TEVAR infection

## Abstract

A single-stage extensive aortic replacement is necessary for infected stent graft explantation after thoracic endovascular aortic repair (TEVAR). However, establishing selective cerebral perfusion and antegrade myocardial protection while ensuring a clear view of the distal aorta is challenging. We adopted a semi-clamshell approach with rib cross for a case of infection following hybrid TEVAR. This approach provides visualization of the ascending aorta, aortic arch, and the descending aorta down to the diaphragm. It is a viable option for cases requiring total arch and distal descending aorta replacement, offering reliable cerebral and myocardial protection, particularly in open conversion of hybrid TEVAR.

## Introduction

The incidence of post-thoracic endovascular aortic repair (TEVAR) infections is reported to be approximately 1.5%–4.8%,^1)^ and among these, approximately 10% occur after hybrid TEVAR.^2)^ In such cases, the stent graft is typically deployed at or beyond zone 2, and complete explantation inevitably requires concomitant total arch replacement.

However, performing myocardial and cerebral protection from a left thoracotomy—commonly used for descending aortic replacement—poses significant technical challenges due to limited access and anatomical constraints.

Herein, we report a case of stent graft infection following 1-branch debranching TEVAR that was successfully treated with a single-stage total arch and descending aortic replacement using a semi-clamshell approach with rib-cross. This approach offered excellent exposure from the ascending aorta to the descending thoracic aorta and enabled complete graft explantation and in situ reconstruction.

## Case Report

**Fig. 1:**
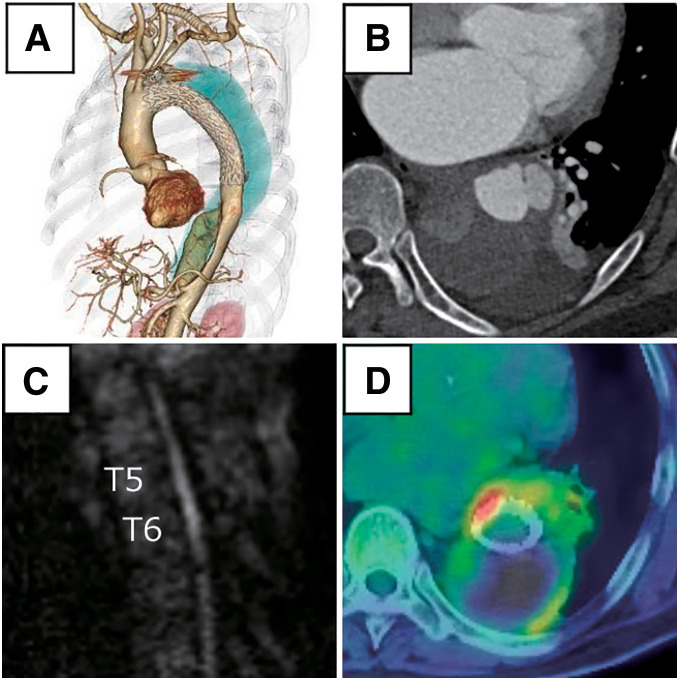
Preoperative imaging findings demonstrating stent graft infection and spinal cord infarction. (**A**) Preoperative 3-dimensional CT showing an aortic aneurysm. (**B**) Contrast-enhanced CT revealing an infected saccular aneurysm (77 × 55 mm) at the distal edge of the stent graft. (**C**) Diffusion-weighted magnetic resonance imaging showing high signal intensity in the spinal cord at the T5–T6 level, consistent with acute spinal cord infarction. (**D**) FDG-PET/CT demonstrating intense uptake along the stent graft and surrounding aneurysmal wall, indicative of infection. CT: computed tomography; FDG: ^18^F-fluorodeoxyglucose; PET: positron emission tomography

A man in his 60s with a history of DeBakey type IIIb chronic aortic dissection had previously undergone 1-branch debranching TEVAR for a dissecting aortic aneurysm (**Fig. 1A**). One year later, he developed sudden-onset paraplegia and fever and was referred to our institution. Computed tomography (CT) imaging demonstrated enlargement of the aortic arch aneurysm and a newly developed saccular pseudoaneurysm, measuring 77 × 55 mm, at the distal end of the stent graft (**Fig. 1B**). Magnetic resonance imaging (MRI) of the thoracic spine showed high signal intensity in both diffusion-weighted and T2-weighted sequences (**Fig. 1C**), consistent with acute spinal cord infarction. The artery of Adamkiewicz, previously identified, remained patent, and no new occlusions of intercostal arteries were observed.

^18^F-fluorodeoxyglucose-positron emission tomography/CT (PET/CT) revealed intense uptake along the entire length of the stent graft, including at the coil placed at the origin of the left subclavian artery, suggesting active infection. The maximum standardized uptake value was 10.98 (**Fig. 1D**), supporting the diagnosis. Laboratory studies showed elevated inflammatory markers, with a white blood cell count of 12200/μL and C-reactive protein of 14.6 mg/dL. Blood cultures were positive for methicillin-sensitive *Staphylococcus aureus*.

To prevent impending rupture of the infected aneurysm, an additional distal TEVAR was performed as a bridging intervention, extending coverage beyond the pseudoaneurysm. Definitive surgical repair was scheduled and performed 1 week later.

**Fig. 2:**
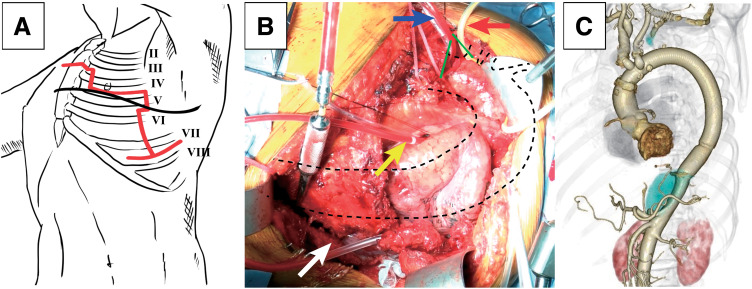
Semi-clamshell approach with rib-cross: surgical technique, intraoperative view, and postoperative outcome. (**A**) Schematic illustration of the surgical approach. The black line indicates the skin incision, and the red line shows the thoracotomy and rib/sternal transection, highlighting the semi-clamshell approach with rib-cross modification. (**B**) Operative field. The aorta is marked with a black dotted line, and the rib-cross is indicated by a white arrow. The cardioplegia cannula (blue arrow) is inserted through the gap created by the sternal transection (green lines). In this operative field, access to the ascending aorta would not have been possible without sternal transection. The yellow arrow indicates the left atrial vent, and the red arrow highlights the taping of the brachiocephalic artery. (**C**) Postoperative 3-dimensional CT. The image shows no evidence of residual infection or graft-related complications. CT: computed tomography

The operation was conducted using a semi-clamshell approach with rib-cross. The patient was placed in a right half-lateral decubitus position, with the upper body close to the lateral position. A left anterior thoracotomy was made along the 4th intercostal space, and the sternum was transected at the level of the 3rd intercostal space. The skin incision was extended posteriorly along the inferior border of the scapula. Both the latissimus dorsi and serratus anterior muscles were divided. The left internal thoracic artery was ligated and divided, while the right internal thoracic artery was preserved. To obtain sufficient visualization of the distal thoracic aorta, the 5th, 6th, and 7th ribs were transected at the posterior axillary line (**Fig. 2A**). This modification provided excellent exposure extending from the ascending aorta to the descending thoracic aorta near the diaphragm (**Fig. 2B**).

Cardiopulmonary bypass was established via the right femoral artery and vein, and a left atrial vent was inserted through the left upper pulmonary vein. After systemic cooling to 25°C, circulatory arrest was induced, and selective antegrade cerebral perfusion was initiated via the brachiocephalic and left common carotid arteries to ensure reliable cerebral protection. The ascending aorta was clamped, and antegrade cardioplegia was administered for myocardial protection.

A 4-branched Dacron graft was used to reconstruct the aorta. The infected stent graft was completely removed, and extensive debridement of surrounding infected tissue, including PET-positive regions, was meticulously performed. Proximal anastomosis was performed at the ascending aorta, followed by reconstruction of the supra-aortic branches. The graft was routed posterior to the phrenic nerve, and the operative field was shifted to the 7th intercostal space to perform the distal anastomosis to the true lumen after trimming the aortic stump at the T11 level. Preoperative imaging demonstrated the Adamkiewicz artery arising from the right T10 intercostal artery. The artery was occluded during the 2nd TEVAR when the stent graft was extended to the inferior border of T10. As the T10 level corresponded to the boundary of the infected area, radical debridement was prioritized, and reconstruction of the Adamkiewicz artery was not performed. Cardiopulmonary bypass was weaned uneventfully. The omentum was not utilized, as it could not adequately cover the entire graft. The sternum and 3 ribs were fixed using a sternal plating system (SternalPlate; Stryker, Kalamazoo, MI, USA). The total operative time was 506 minutes. Cardiopulmonary bypass time was 286 minutes, aortic cross-clamp time was 231 minutes, and selective cerebral perfusion time was 140 minutes. The postoperative course was smooth, with gradual improvement of paraplegia. Postoperative CT confirmed that the infected stent graft and aneurysm of the aorta had been completely resected (**Fig. 2C**). The patient was extubated 31 hours after surgery and discharged home ambulatory following 2 months of rehabilitation. Although the exact cause of paraplegia remained uncertain, it was presumed to result from microemboli or compression of collateral circulation caused by the infected aneurysm. Intravenous antibiotics were administered for 4 weeks postoperatively and subsequently discontinued. No recurrence of infection was observed at 18 months postoperatively.

## Discussion

Inherently, open descending aortic replacement is a technically demanding procedure, and it has become increasingly uncommon with the rise of TEVAR, resulting in further limited surgical experience. The variety of exposure techniques adds further complexity to standardization. Despite this trend, open surgery remains essential in cases of stent graft infection. These procedures are often technically demanding and performed in anatomically challenging settings. The semi-clamshell approach with rib-cross ensures adequate exposure of the proximal, arch, and distal segments to meet the specific operative requirements at each level, and is therefore particularly beneficial in the surgical management of stent graft infection. The requirements at each level are as follows.

At the proximal level, infection control surgery requires direct access to the ascending aorta in a single-stage procedure. Traditional 2-stage approaches involving median sternotomy followed by left thoracotomy may be insufficient. Accessing the ascending aorta through a left thoracotomy mandates a surgical field that also allows for safe myocardial protection.

At the arch level, procedures in infected fields are typically prolonged. Techniques relying on profound hypothermia and retrograde cerebral perfusion through elevated CVP, such as the Takamoto method,^3)^ may become insufficient in prolonged procedures. Hence, selective antegrade cerebral perfusion is preferred for its reliability. This necessitates clear visualization of the arch vessels for both perfusion and reconstruction.

At the distal level, extensive arch and descending thoracic aortic replacement is particularly challenging due to tissue fragility and the likelihood of needing more extensive resection than preoperatively anticipated. Even in the absence of a clear pseudoaneurysm, infection may weaken the distal landing zone, requiring reconstruction down to the level just above the diaphragm. In such cases, sufficient exposure of the distal thoracic aorta is essential.

**Fig. 3:**
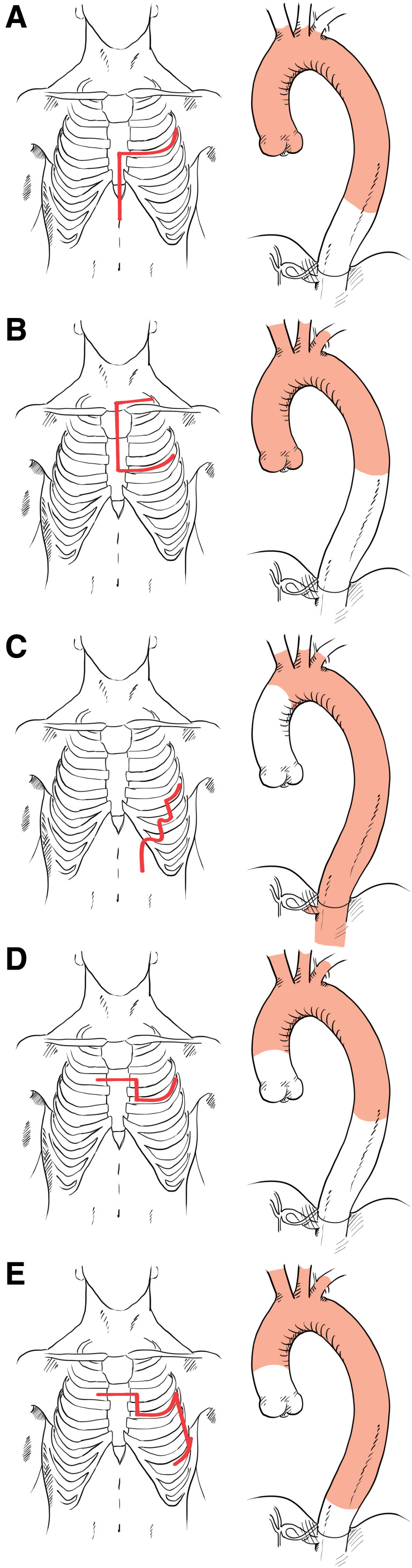
Major surgical approaches with skin incisions and extent of aortic replacement. (**A**) ALPS, (**B**) trap-door, (**C**) SIRC, (**D**) semi-clamshell, and (**E**) semi-clamshell with rib-cross. ALPS: anterolateral thoracotomy with partial sternotomy; SIRC: straight incision with rib-cross

Several alternative surgical approaches have been described to meet these anatomical demands. Anterolateral thoracotomy with partial sternotomy involves a partial sternotomy combined with a left thoracotomy and provides access similar to that used in routine cardiac surgery, enabling aortic root operations and myocardial protection. Although the distal descending aorta appears deeper near the diaphragm when viewed from the anterior direction, previous reports have described successful distal anastomoses as far as T10–T12 (**Fig. 3A**).^4)^ The trap-door approach, which extends a median sternotomy with a transverse incision at the 4th intercostal space and a supraclavicular extension, is another option. While this technique provides poorer exposure of the distal descending aorta, it offers excellent visualization of the supra-aortic branches (**Fig. 3B**).^5)^ Straight incision with rib-cross involves a straight incision from the axilla to the costal margin.^6)^ It allows exposure of the supra-aortic branches even in patients with thin chest walls; although it limits manipulation of the ascending aorta due to its lateral approach, it is useful in cases requiring replacement extending to the thoracoabdominal aorta (**Fig. 3C**).

Furukawa et al. previously reported the use of a semi-clamshell approach involving transverse sternotomy and left anterior thoracotomy, enabling replacement from the ascending aorta to the T7 or T8 level while reducing the invasiveness compared to full clamshell incisions (**Fig. 3D**).^7)^ Although there are reports of ascending aortic anastomosis without sternal transection,^8)^ this is particularly challenging in patients with narrow thoracic anatomy, such as those in East Asia. Therefore, the semi-clamshell approach represents a reasonable method for accessing the ascending aorta. We extended Furukawa’s approach by adding posterior transection of the 5th through 7th ribs, enabling exposure further down to the diaphragm (**Fig. 3E**).

All of the above approaches can be considered options for managing TEVAR infection; however, we believe that the semi-clamshell approach with rib-cross offers a well-balanced solution. This method not only provides adequate exposure of the proximal, arch, and distal segments without undue difficulty but also facilitates bleeding control. In infected cases, wide dissection is often required, and persistent bleeding from fragile tissue planes may occur after weaning from cardiopulmonary bypass. The semi-clamshell with rib-cross offers stable visualization of the descending aorta even after bypass weaning, making it particularly suitable for infected cases that require extensive debridement and reliable hemostasis.

To complement this approach, myocardial protection was achieved with Del Nido cardioplegia, which provided prolonged protection, simplified dosing, and was particularly advantageous during the open proximal phase with limited visibility.^9)^

Despite these advantages, the semi-clamshell approach requires caution regarding respiratory complications. Prior studies have reported a tracheostomy rate of 17% following full clamshell incisions.^10)^ The semi-clamshell, using left thoracotomy alone, may reduce this risk. Nonetheless, reducing operative time is crucial in minimizing pulmonary complications. In reports where the average surgical duration exceeded 9 hours, 53% of patients experienced delayed ventilator weaning.^7)^ Secure fixation of the sternum and ribs is also essential.

Preoperative imaging plays a crucial role in surgical planning. In particular, the levels of intercostal thoracotomy and sternal transection are difficult to modify intraoperatively. Therefore, careful preoperative assessment of the height of the supra-aortic branches is recommended. In our case, the most cranial branch arose at the level of the 1st intercostal space, allowing a favorable angle for anastomosis. Moreover, the distance between the ascending aorta and the posterior sternum was 2 cm on preoperative CT, which was expanded to approximately 4 cm intraoperatively by decompression of the aorta and elevation of the sternal transection, ensuring a safe proximal anastomosis. For cases with minimal aortic-sternal distance or high-riding supra-aortic branches, a left thoracotomy approach should be considered with caution.

## Conclusion

We successfully treated a case of infected stent graft following hybrid TEVAR using single-stage total arch and descending thoracic aortic replacement through a semi-clamshell approach with rib-cross. This technique provided excellent exposure, enabled complete graft explantation, and allowed safe proximal and distal reconstruction with effective organ protection. Tailoring the surgical approach to patient anatomy, disease extent, and operative goals is essential. Our experience suggests this modified technique is a valuable option in managing complex aortic infections.

## Declarations

### Data availability

The datasets used and analyzed during the current study are available from the corresponding author upon reasonable request.
